# The Ketogenic Diet Through a Metabolomic Lens: Biochemical Pathways, Therapeutic Applications, and Analytical Challenges

**DOI:** 10.3390/nu17182969

**Published:** 2025-09-16

**Authors:** Katarzyna Idzikowska, Paulina Gątarek, Anna Gajda, Piotr Safiński, Lukasz Przyslo, Joanna Kałużna-Czaplińska

**Affiliations:** 1 Institute of General and Environmental Chemistry, Faculty of Chemistry, Lodz University of Technology, 116 Zeromskiego Street, 90-924 Lodz, Poland; 2 Institute of Organic Chemistry, Faculty of Chemistry, Lodz University of Technology, 116 Zeromskiego Street, 90-924 Lodz, Poland; anna.gajda@p.lodz.pl; 3 Department of Developmental Neurology and Epileptology, Research Institute of Polish Mother’s Memorial Hospital, 93-338 Lodz, Poland; safinski@gmail.com (P.S.); lukasz.przyslo@iczmp.edu.pl (L.P.)

**Keywords:** dietary interventions, metabolic disorders, neurological disorders, chromatography–mass spectrometry techniques

## Abstract

Background: The ketogenic diet (KD), a high-fat and low-carbohydrate dietary approach, has been used therapeutically in drug-resistant epilepsy and other neurological and metabolic disorders. Recent interest has shifted toward understanding its broader metabolic effects through metabolomics. This review aims to summarize current knowledge on the biochemical mechanisms and therapeutic implications of the KD, with a particular focus on metabolomic profiling and neurological health. Methods: This narrative review synthesizes findings from the last five years of metabolomic studies investigating the biochemical consequences of the KD and its variants, including the classical KD, modified Atkins diet (MAD), medium-chain triglyceride diet (MCT), and low glycemic index treatment (LGIT). The review integrates data on analytical techniques, such as liquid chromatography–mass spectrometry (LC-MS) and gas chromatography–mass spectrometry (GC-MS), and evaluates alterations in key metabolic pathways. Results: The KD significantly modulates energy metabolism, shifting adenosine triphosphate (ATP) production from glycolysis to fatty acid oxidation and ketone body utilization. It affects mitochondrial function, one-carbon metabolism, redox balance, neurotransmitter regulation, and gut–brain axis signaling. Metabolomic profiling has identified β-hydroxybutyrate (βHB) as a key regulatory metabolite influencing mitochondrial respiration. Long-term KD use may impact renal and hepatic function, necessitating clinical caution and individualized nutritional monitoring. Conclusions: Metabolomic analysis provides critical insights into the multifaceted effects of the KD, supporting its role as a targeted metabolic therapy in neurological diseases. However, potential risks linked to prolonged ketosis warrant further investigation. Future studies should focus on personalized applications and long-term safety profiles of KD variants across patient populations.

## 1. Introduction

The ketogenic diet (KD) is a nutritional model characterized by high fat content, moderate protein intake, and very low carbohydrate consumption, which leads to a marked reduction in glucose availability as the primary energy source [1]. As a result, the body enters a state of ketosis, in which ketone bodies—mainly β-hydroxybutyrate (βHB), acetoacetate, and acetone—produced through enhanced β-oxidation of fatty acids in the liver, become the predominant substrates for cellular energy metabolism [2]. This metabolic adaptation fundamentally reprograms the pathways of energy production, mitochondrial function, and redox balance, while also influencing neurotransmitter homeostasis and inflammatory signaling [3].

The KD should primarily be regarded as a therapeutic intervention rather than a conventional dietary approach. Its application is rooted in clinical contexts, most notably in the management of drug-resistant epilepsy, where its efficacy has been documented for nearly a century [4]. More recently, the KD has been investigated as a potential adjunctive therapy in metabolic disorders, neurodegenerative diseases, and certain cancers, highlighting its role as a medical treatment strategy rather than a lifestyle or nutritional choice [5,6]. This therapeutic perspective underscores the importance of differentiating KD from popular dietary trends and recognizing both its clinical benefits and risks within the framework of evidence-based medicine.

Originally developed for epilepsy, particularly in children, the KD remains an important clinical tool in neurology [7]. Over time, clinical and experimental data have expanded its potential applications, with research exploring its use in conditions such as Alzheimer’s disease, Parkinson’s disease, obesity, type 2 diabetes, metabolic syndrome, and cancer [5,6,8,9,10]. The resurgence of interest in the KD has also contributed to its popularization among the general public, where it is sometimes controversially promoted as an “optimal” diet. However, when applied outside medical supervision, this approach raises concerns about potential adverse effects [7].

At the mechanistic level, the therapeutic potential of the KD derives from its ability to induce ketosis and thereby shift cellular energy metabolism from glucose to ketone utilization [2]. Beyond serving as alternative fuels, ketone bodies—especially βHB—act as signaling metabolites that regulate gene expression, epigenetic modifications, oxidative stress, and inflammatory pathways [11]. The KD has also been shown to modulate gut microbiota, altering microbial composition and metabolite production, which may further contribute to its systemic and neurological effects.

The investigation of these mechanisms has been greatly advanced by metabolomics, which enables comprehensive profiling of metabolic alterations associated with the KD [9]. By employing techniques such as liquid chromatography–mass spectrometry (LC-MS), gas chromatography–mass spectrometry (GC-MS), and nuclear magnetic resonance spectroscopy (NMR), metabolomics allows precise quantification of metabolites and the identification of biomarkers linked to ketosis, therapeutic efficacy, and neurological outcomes [2,9]. Such approaches not only deepen our understanding of the KD’s biochemical effects but also provide tools for individualized patient monitoring and optimization of dietary protocols.

Clinically, the KD has shown robust efficacy in reducing seizure frequency in drug-resistant epilepsy, with benefits documented in both pediatric and adult populations [4,7]. Its therapeutic promise has extended to metabolic diseases, where it improves insulin sensitivity, reduces visceral adiposity, and lowers glycated hemoglobin (HbA1c) levels [1]. In neurodegenerative disorders, ketone bodies may compensate for impaired glucose metabolism in the brain, while exerting neuroprotective effects through improved mitochondrial efficiency and reduced oxidative stress [6,10]. Preliminary studies in oncology further suggest that the KD could enhance therapeutic efficacy by exploiting the metabolic vulnerabilities of tumor cells [5,9].

Despite this broad therapeutic potential, the long-term safety and sustainability of the KD remain controversial. Reported side effects include dyslipidemia, nephrolithiasis, gastrointestinal discomfort, micronutrient deficiencies, and, in children, impaired growth [3,7]. Moreover, adherence to the restrictive macronutrient profile of the KD poses a significant challenge for patients, often limiting its applicability outside specialized clinical settings. These limitations highlight the need for careful medical supervision, targeted supplementation, and further research into patient-specific responses.

In this context, metabolomic studies are uniquely positioned to clarify both the benefits and risks of the KD, offering a systems-level perspective on its multifaceted effects. By integrating metabolomic data with clinical outcomes, researchers can identify biomarkers predictive of efficacy and safety, stratify patients most likely to benefit from dietary intervention, and refine therapeutic strategies.

**Figure 1:**
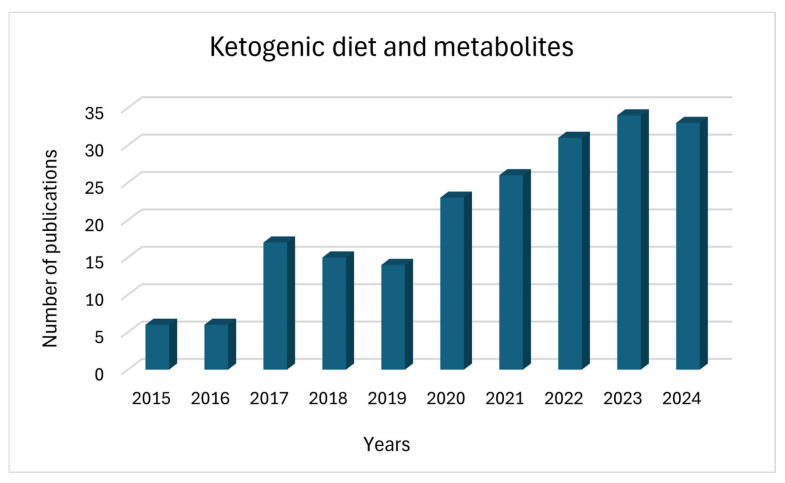
Trends in the number of publications on the ketogenic diet (KD) involving metabolite analysis during the last decade (2015–2024), according to PubMed.

The present review aims to provide a comprehensive and critical evaluation of recent clinical and preclinical studies on the KD and its therapeutic variants, with a particular focus on neurological disorders. Besides summarizing established clinical outcomes, we emphasize how metabolomic and complementary high-throughput approaches—such as LC-MS, GC-MS, NMR, magnetic resonance spectroscopy/imaging (MRS/MRI), and stable isotope tracing—have advanced our understanding of KD-induced biochemical and physiological adaptations. These technologies allow precise mapping of metabolic remodeling, including alterations in lipid and amino acid pathways, ketone body fluxes, mitochondrial function, and neurotransmitter metabolism. By integrating these insights with clinical data, metabolomics not only describes the metabolic shifts elicited by the KD but also identifies predictive and mechanistic biomarkers that support a more personalized and safe application of this dietary therapy. In this context, metabolomics has transformed the understanding of the KD from a descriptive dietary intervention into a mechanistically defined therapy, providing both explanatory and predictive insights. Recently, there has been a significant increase in interest in the KD. On the Pub-Med platform, 5581 articles have been published in the time frame 1931–2025. However, more than 50% of these publications were written in the last five years. There are far fewer papers combining the topic of KD and metabolite research—only 226 articles between 1979 and 2025—and more than 64% of these were written in the last five years (Figure 1).

The literature review was conducted based on an analysis of scientific articles related to the ketogenic diet, metabolism, and the application of chromatographic techniques in their investigation. The focus was primarily on publications from the past five years. The literature was selected using search results from the Google Scholar https://scholar.google.com (accessed on 10 February 2025) and PubMed https://pubmed.ncbi.nlm.nih.gov (accessed on 13 February 2025) databases. The following keywords were used: ketogenic diet, ketogenic diet and metabolomics, ketogenic diet and metabolism, ketogenic diet and chromatographic techniques.

**Figure 2:**
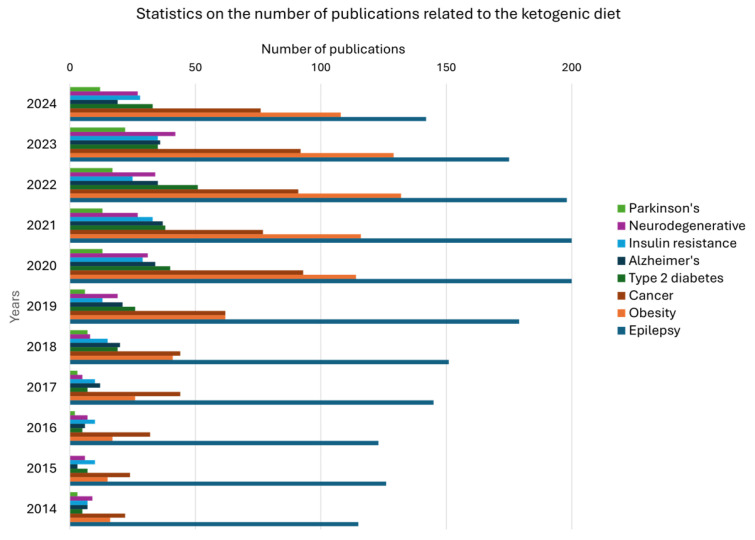
The number of publications on the use of the KD in various diseases.

In recent years, there has been a dynamic increase in interest in the KD in the context of various diseases, which is reflected in the number of scientific publications available on the PubMed platform, presented in Figure 2. For the term “epilepsy and KD”, the first significant increase in the number of scientific papers was observed as early as 1998, when the number of publications rose from 10 to 20 within a year, and as many as 61% of all 2365 papers have been published in the last 10 years. In the case of the term “obesity and KD”, the number of publications grew steadily from 2003 to 2013—from 4 to 20—and 84% of the 813 results come from the last decade. The term “type 2 diabetes and KD” is characterized by an even greater concentration of current research—over 91% of all 261 publications have appeared in the last 10 years. A similar trend can be seen for the term “insulin resistance and KD”, for which there was a sharp increase in the number of articles at the turn of 2019 and 2020 (from 13 to 29), with almost 85% of the 222 results published in the same decade. The increase in interest in the KD in the context of neurodegenerative diseases (“neurodegenerative and KD”) dates back to 2010, with a clear rise between 2018 and 2020—85% of the 222 publications are from the last 10 years. In the area of Parkinson’s disease (“Parkinson and KD”), the number of publications doubled in 2020 (from 6 to 13), and 82% of 99 articles were published in the last decade. In the case of Alzheimer’s disease (“Alzheimer and KD”), the first studies appeared in 2005, and between 2017 and 2020, there was a sharp increase in publications—from 12 to 34—resulting in as many as 89% of 220 studies being published in the last 10 years. The topic of the relationship between the KD and cancer (“cancer and KD”) has been present in the literature since 1980, but the greatest interest was in the years 2017–2020, when the number of publications increased to 93; over 85% of the 667 results were published in the last decade. These data clearly indicate the growing importance of the KD in research on a wide range of diseases and health disorders.

## 2. Milestones of the KD

Ketogenic therapy, understood today as a medical intervention using the effects of the state of ketosis on the pathophysiology of selected diseases, has its roots going back to antiquity. Already in medical texts attributed to Hippocrates of Kos (c. 460–370 BC) and his disciples and successors—collected in the so-called Corpus Hippocraticum—there are references to the therapeutic use of fasting in the treatment of epileptic seizures [12,13,14].

The first modern reports on the use of starvation in the treatment of epilepsy date back to the early 20th century. In 1911, two French physicians, Guelpa and Marie, published the results of a clinical observation in which they applied fasting to 26 epileptic patients. Six of them showed a marked reduction in seizure frequency and severity [14,15].

A more detailed report on the therapeutic use of fasting in the treatment of epilepsy was presented in 1921 by Geyelin at the congress of the American Medical Association. The results of a controlled study conducted on a group of 36 patients showed a clear reduction in the number of seizures—by up to 80% [16].

Despite such promising results, fasting as a therapeutic method had a significant limitation—its long-term use was not feasible for physiological and practical reasons. Around the same time, research began into the possibility of nutritional control of hyperglycemia in diabetic patients. Based on the earlier work of Newburgh and Marsh, Woodyatt in 1921 proposed the use of high-fat and low-carbohydrate diets as a method of regulating blood glucose levels. He also observed that both increased dietary fat supply and fasting in healthy individuals lead to the onset of controlled ketosis, a condition in which the body, adapting to the limited availability of glucose, begins to use fats as the main source of energy. The physiological consequence of this process is an increase in the concentration of ketone bodies in the blood, such as (R)-β-hydroxybutyric acid, acetoacetic acid (AcAc), and acetone [17,18].

In the same year, Wilder suggested that the beneficial effect of fasting on the reduction of epileptic seizures could be directly related to the induction of a state of ketosis. He proposed replacing fasting with a more sustainable method of achieving ketosis—by following a high-fat, low-carbohydrate diet, which he called the “ketogenic diet”. The dietary protocol developed by Peterman called for an intake of 1 g of protein per kilogram of body weight, 10–15 g of carbohydrates per day, and the remainder of the caloric requirements to be covered by fat. This protocol, in an almost unchanged form, remains the basis of the classic KD used to this day [19,20,21,22].

The 1920s and 1930s were a period of intensive use of the KD in clinical practice as a treatment for epilepsy. However, with the discovery and spread of new antiepileptic drugs—such as diphenylhydantoin (1938) and valproic acid (1967)—the importance of the KD gradually declined. In the following decades, it was mainly used in the treatment of drug-resistant epilepsy, especially in children with unsatisfactory drug treatment.

The renaissance of interest in the KD occurred in the 1990s and was linked to the media-published story of Charlie, the son of film director Jim Abrams. A film documenting the effectiveness of ketogenic therapy in treating the boy’s severe drug-resistant epilepsy and the establishment of the Charlie Foundation provided the impetus for a resurgence of extensive research into the KD—in the context of treating not only epilepsy but also other neurological and metabolic diseases.

**Figure 3:**
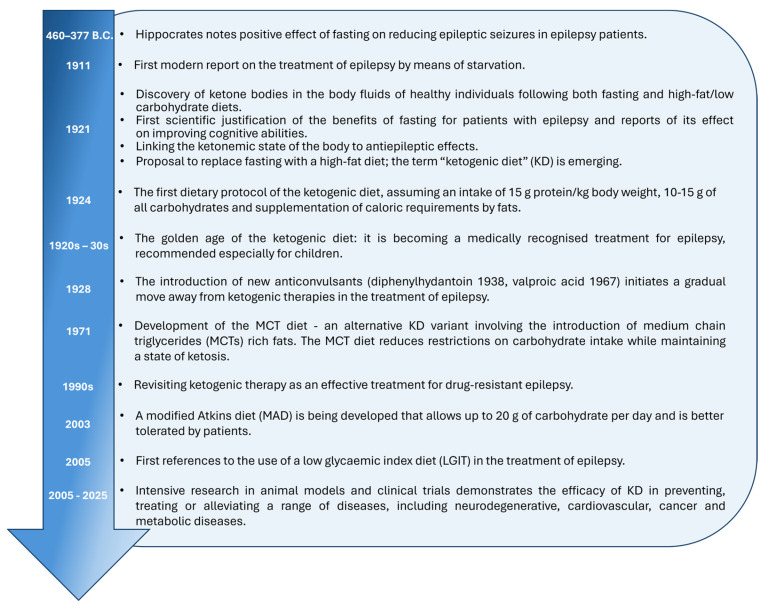
Milestones of the KD [13,14,22,28,29,30].

Since Wilder’s introduction of the classic KD in 1921, a number of modifications to the diet have been developed to increase nutritional flexibility and better adapt to patients’ needs. In 1971, Huttenlocher and co-workers proposed the MCT, based on fats containing medium-chain fatty acids—mainly octanoic and decanoic acid—which show greater ketogenic potential [23]. In 2003, the MAD was developed, and in 2005 [24,25,26], LGIT, based on the consumption of low glycemic index (GI < 50) foods, was introduced [27]. A summary of the historical overview is presented in Figure 3.

### 2.1. Classic Ketogenic Diet

There are various approaches to the classic ketogenic diet (CKD). The standard CKD is a 3:1 diet. The CKD is the oldest and most thoroughly researched form of the KD. Extreme ketogenic therapy or therapy used in the initial phase of treatment to quickly achieve ketosis is based on a 4:1 ratio of fat to protein and carbohydrates in the diet, i.e., 4 g of fat for every 1 g of protein and carbohydrates. The 4:1 ratio derives about 90% of the total energy from fat, 6% from protein, and 4% from carbohydrates, which ensures a high level of ketosis. A slightly more flexible variation of the 3:1 ratio is characterized by a reduction in fat content to 87%, with protein and carbohydrates accounting for 10% and 3% of the diet, respectively. This ratio of the CKD is most often used during developmental age because it provides additional protein intake while maintaining control of epileptic seizures. The CKD is highly effective in reducing drug-resistant seizures, but requires strict adherence to dietary and supplementation recommendations, as it is associated with potential side effects such as gastrointestinal symptoms and hyperlipidemia. Close monitoring with regular check-ups and clinical assessment is essential to ensure the safety of the therapy and optimize results. The implementation of a classic ketogenic diet in children, especially infants, always requires hospitalization. Furthermore, fasting, as one of the ways to achieve ketosis, is strictly contraindicated in the youngest children and in patients with mitochondriopathies. Other forms of the ketogenic diet can be implemented on an outpatient basis in close cooperation between the patient, neurologist, and clinical dietitian [31].

### 2.2. Modified Atkins Diet

The MAD is a more flexible version of the CKD. The ketogenic ratio in the MAD is usually 2:1, similar to the CKD, and consists of approximately 82% fat, 12% protein, and 6% carbohydrates, which allows for moderate ketone body concentrations while improving diet compliance. A modified version of the MAD can be an even less restrictive variation of the 2:1 version and maintain a composition of 77% fat, 17% protein, and 6% carbohydrates, which further facilitates patient compliance. Unlike the CKD, the implementation of the MAD does not require hospitalization, making it suitable for older children, adolescents, and adults. Daily carbohydrate intake is limited to 10–20 g in children and 25 g in adults, with unlimited protein intake. In terms of anti-seizure efficacy, it has an effect similar to the CKD and is associated with fewer side effects [31].

### 2.3. Medium-Chain Fatty Acid Diet

The MCT diet involves the use of medium-chain triglycerides, which usually come from sources such as coconut oil and palm oil. Medium-chain triglycerides are more easily absorbed and converted into ketones, allowing for higher carbohydrate and protein intake than in the classic KD. In this version, 70% of total energy comes from fat, 10% from protein, and 15–19% from carbohydrates, with 30–60% of fat intake provided by MCT oil. Enriching the KD with MCTs, along with increased supplementation, is beneficial in cases of hypercholesterolemia. However, increased amounts of MCT oil in the diet are often associated with diarrhea and bloating and are therefore not preferred in the youngest patients [31].

### 2.4. Low Glycemic Index Diet and Low-Carbohydrate Diet

LGIT involves maintaining blood glucose levels by limiting carbohydrate intake to foods with a low glycemic index (GI < 50) rather than adhering to a strict ketogenic ratio. LGIT offers a more balanced macronutrient distribution of 60% fat, 30% protein, and 10% carbohydrates, with a daily carbohydrate intake of 40–60 g. The LGIT diet is significantly less restrictive than other ketogenic variants and is an ideal choice for patients who have not followed dietary recommendations or have experienced severe side effects from the CKD. LGIT will not result in high ketone concentrations, but it is effective in preventing seizures and ensures positive metabolic control, which may be important in patients with drug-resistant epilepsy and metabolic syndrome [31]. A summary of the main principles of the aforementioned types of ketogenic diets and a comparison of regular diet with the classic KD is presented in Table 1.

## 3. The Metabolic Perspective in the Context of a KD

### 3.1. Energy Pathway: ATP and Krebs Cycle

The KD is a nutritional intervention characterized by a marked reduction in carbohydrate intake and an increase in fat consumption, leading to profound alterations in energy metabolism, particularly in ATP production, β -oxidation, and mitochondrial function [32,33]. Reduced glucose availability inhibits glycolysis and decreases pyruvate levels, limiting oxaloacetate production, which not only diminishes substrate entry into the Krebs cycle (TCA) but also redirects oxaloacetate to gluconeogenesis under low-carbohydrate conditions, a process known as cataplerosis [34,35,36,37]. Consequently, acetyl-CoA derived from intensified β-oxidation of fatty acids exceeds the TCA’s capacity and is diverted to ketogenesis, producing acetoacetate, βHB, and acetone in the liver [34,35,37,38]. These ketone bodies circulate to peripheral tissues, including the brain, heart, and skeletal muscle, where they are converted back to acetyl-CoA via enzymes such as SCOT and βHB dehydrogenase, entering the TCA in tissues with adequate oxaloacetate availability to sustain NADH, FADH_2_, and ATP production [34,35,39]. Over time, mitochondria adapt by increasing in number and enhancing oxidative phosphorylation, while the carnitine pathway facilitates the transport of long-chain fatty acids into the mitochondrial matrix to support ongoing β-oxidation [33,39]. The KD also modulates the endocrine system by reducing insulin secretion, which decreases HMG-CoA reductase activity, contributing to improved lipid profiles, including reduced triglycerides [40,41]. Small amounts of glucose are still formed through gluconeogenesis from proteins and glycerol, slowing glycogenesis [33,36]. While short-term ketosis enhances mitochondrial efficiency and provides a preferred energy source for tissues such as the brain—where βHB can supply 60–70% of energy—and cardiomyocytes, long-term KD may induce progressive depletion of TCA intermediates, necessitating anaplerotic mechanisms via amino acid metabolism and increased pyruvate carboxylase activity [37,39,42]. Persistent cataplerosis may impair biosynthesis of non-essential amino acids and other anabolic compounds, and in certain cell types, prolonged exposure to high ketone concentrations could reduce mitochondrial biogenesis and increase apoptosis susceptibility, highlighting the need for careful monitoring during long-term KD [35,39,43]. Overall, KD transforms energy metabolism by suppressing glycolysis and stimulating β-oxidation, ketogenesis, and mitochondrial oxidative phosphorylation, leading to increased ATP production, improved redox balance, and multifaceted metabolic and neurological effects [32,40,44].

### 3.2. One-Carbon Metabolism 1C

The one-carbon pathway is a complex network of biochemical reactions involving three major metabolic cycles: the folate cycle, the methionine cycle, and the transsulphuration pathway. These interconnected pathways play a key role in essential cellular processes such as DNA and RNA synthesis, protein and histone methylation, regulation of gene expression, and maintenance of redox homeostasis. The main functions of the pathway include the supply of methyl groups for DNA methylation, the synthesis of nucleotides (particularly purines and thymidine), and the remethylation of homocysteine to methionine [45]. Changes in the availability of substrates, such as glucose and glucogenic amino acids, can indirectly affect the function of the one-carbon pathway. Reducing the availability of glucose affects the availability of precursors for the one-carbon pathway, which may alter the balance between different metabolic pathways [46].

Key components of the pathway include methyl group donors (folate, methionine, choline, betaine), enzyme cofactors (vitamins B2, B6, B12, zinc), and major metabolites (S-adenosylmethionine (SAM), homocysteine, formate) [45,47]. The one-carbon pathway requires a constant supply of nutrients and is highly sensitive to dietary changes [48].

The most important finding regarding the effect of the KD on the one-carbon pathway is the significant inhibition of mitochondrial production of formate, a key carrier of one-carbon units in cells. Studies using a KD enriched in MCT have shown that it leads to inhibition of the activity of the glycine breakdown system (GCS)—the main mitochondrial source of formate. A 19% reduction in the plasma concentrations of formate was observed in mice using MCT-KD. In addition, the KD results in a significant reduction in carbon flux from glycine and serine to formate, whereby glycine is reduced to undetectable levels [49].

βHB, the main ketone produced during ketosis, shows a direct inhibitory effect on the one-carbon pathway [50]. In in vitro studies, βHB at a concentration of 10 mM caused a 55% inhibition of deoxythymidine monophosphate (dTMP) nucleotide synthesis. The mechanism of this action involves a reduction in the availability of one-carbon units for biosynthetic processes and an effect on the activity of the enzyme methylenetetrahydrofolate dehydrogenase 2 (MTHFD2), a key player in the mitochondrial one-carbon pathway [49].

βHB also affects epigenetic processes by modulating DNA methylation and histone acetylation, which may indirectly affect the one-carbon pathway through changes in gene expression. Changes in methylation status are observed in several important genes, whose methylation is particularly associated with weight loss and/or ketosis induced by a very low-carbohydrate ketogenic diet (VLCKD). These genes include ZNF331, FGFRL1, CBFA2TC3ORF38, C3ORF38, JSRP1, and LRFN4 [50]. The KD increases levels of adenosine, which in turn inhibits DNA methylation. This suggests that the effect of the KD on adenosine metabolism may be an indirect but key point of contact with the one-carbon pathway, as adenosine affects the levels of SAM, the main donor of methyl groups [46,50].

The KD also has a significant effect on the biosynthesis of methionine, a key amino acid in the one-carbon pathway. In the liver of mice on MCT-KD, a 48% decrease in methionine synthesis from the mitochondrial-derived formate was observed. In addition, the contribution of the mitochondrial-derived formate to methionine synthesis (5-methyltetrahydrofolate-dependent) significantly reduced in the liver, by 23% [49].

Particular attention should be paid to the effect of the KD on homocysteine homeostasis. Deficiencies in components of the mono-carbon pathway, particularly folate and vitamin B12, result in disturbances in the methylation cycle, leading to homocysteine accumulation. High levels of homocysteine are an independent risk factor for many diseases, including neurodegenerative diseases, stroke, and cardiovascular problems. Research indicates that homocysteine is a marker of disruption in the one-carbon pathway rather than a direct cause of disease. Elevated homocysteine can lead to oxidative stress, mitochondrial dysfunction, and neuronal death [45,51].

In the context of vulnerable populations, women of childbearing age should be particularly cautious about the long-term use of a KD. The mono-carbon pathway is crucial for normal fetal development, and folate deficiencies can lead to neural tube defects [52]. Nutritional status, in terms of components of the one-carbon pathway during pregnancy, is crucial for normal fetal development [48].

Strategies to minimize the risks associated with the effects of a KD on the mono-carbon pathway include targeted supplementation of ingredients that support the pathway. Supplementation with folic acid or its active form (5-methyltetrahydrofolate (5-MTHF)), vitamin B12 in the form of methylcarbylamine, vitamin B6 as an enzyme cofactor, and betaine as an alternative methyl group donor may help to maintain the normal function of the mono-carbon pathway [45,52].

**Figure 4:**
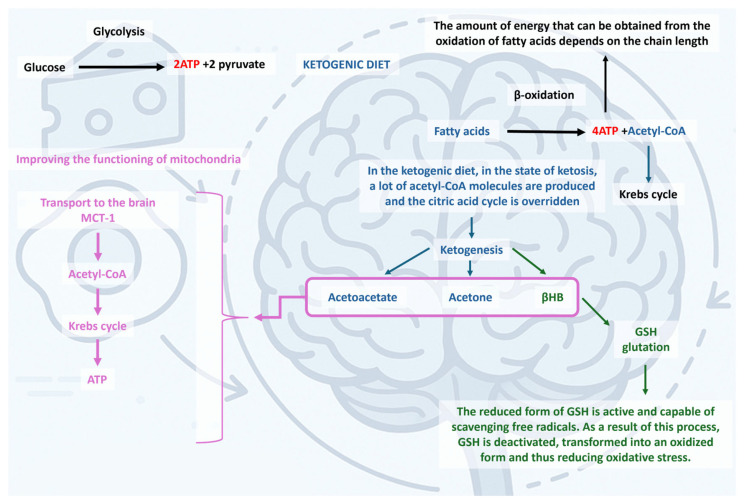
The main metabolic pathways affected by the KD. The differences between energy production in glycolysis and β-oxidation are marked in blue. The differences in the amount of ATP produced in glycolysis and β-oxidation are marked in red. A simplified diagram of the effect of KD on improving mitochondrial function is marked in purple. A simplified diagram of the effect of KD on improving oxidative stress is marked in green.

Below, Figure 4 illustrates the main pathways affected by the KD.

### 3.3. Selected Other Metabolic Pathways Involved in Antioxidant Activity

Low levels of insulin initiate an enzymatic reaction whereby two molecules of acetyl-CoA are converted to acetoacetate, which, in the next step, can decompose to acetone or be metabolically converted to βHB. In addition to its function as an energy substrate, βHB also acts as a signaling molecule influencing mitochondrial function and TCA efficiency [53,54,55]. Studies have shown that βHB improves mitochondrial respiration parameters, increases the stability of the ATP/O_2_ ratio, and reduces the production of reactive oxygen species (ROS). The mechanism of this action includes modulation of calcium transport through mitochondrial channels, which affects the activity of respiratory chain complexes and thus the efficiency of oxidative phosphorylation [55,56].

βHB, as the main ketone metabolite formed during ketosis, is a key factor in modulating redox processes in the body through direct and indirect antioxidant mechanisms. The KD has a significant effect on oxidative stress through a number of related metabolic pathways, in which ketone bodies act directly as free radical scavengers and indirectly modulate redox pathways by increasing the NAD^+^/NADH ratio [57]. The KD reduces the production of ROS by reducing glycolysis and promoting fat and ketone metabolism, thereby alleviating mitochondrial sources of oxidative stress [58]. The KD improves mitochondrial function by, among other things, stimulating mitophagy and inhibiting apoptosis, leading to reduced ROS production and cellular damage [57].

Experimental studies confirm the efficacy of the restrictive KD in reducing oxidative stress, as demonstrated in a four-week study on young rats with diet-induced obesity, where the KD reduced oxidative stress through an increase in the activity of the antioxidant enzyme superoxide dismutase (SOD) and a decrease in malondialdehyde (MDA-a marker of lipid peroxidation) [59]. In vitro, in vivo, and clinical studies show that the KD can reduce oxidative stress, especially in pathological conditions such as brain injury, cancer, or epilepsy, but the effects depend on the time of adaptation and the type of KD used [60]. In the context of cardiovascular diseases, the KD has been shown to have a wide range of mechanisms of action, including effects on reducing inflammation and ROS [61].

The KD also leads to a significant increase in glutathione (GSH) levels in neural tissue, which has been confirmed in both animal studies and clinical observations in patients with epilepsy [62]. The mechanism of this action involves activation of the NRF2 (nuclear factor erythroid 2-related factor 2) transcriptional pathway, which induces the expression of genes encoding antioxidant enzymes, including glutamate-cysteine synthase (GCL) responsible for GSH biosynthesis [63,64]. A study using MRS showed that patients following an MAD with MCT showed up to 7- and 14-fold increases in GSH concentrations in the gray and white matter of the brain, respectively [62]. Long-term use of the KD also leads to increased glutamate and glutamine levels in the cortex and hippocampus, indicating modulation of the glutamate–glutamine cycle between neurons and astrocytes and enhancement of neuroprotective mechanisms [64].

### 3.4. The Gut–Brain Axis and Neurotransmitters

The biochemical mechanisms of the KD in the context of neuroprotection are based on complex interactions between energy metabolism, the composition of the gut microbiome, and neurotransmitter balance in the brain. βHB, as the main metabolite of the KD, is a key signaling molecule that not only modulates neurotransmitter gene expression in the brain, but also has a direct effect on the composition and functionality of the gut microbiome [65].

The KD induces profound changes in the composition of the gut microbiome, increasing the proportion of beneficial bacterial strains such as *Akkermansia muciniphila*, *Bacteroides*, and *Bifidobacterium*, while reducing populations of proinflammatory bacteria [66,67]. Modulation of the gut microbiome by the KD leads to increased production of short-chain fatty acids such as butyrate, propionate, and acetate, which serve as additional energy sources for colonocytes and support the integrity of the intestinal barrier by stimulating epithelial regeneration. These modifications of the gut microbiota translate directly into the regulation of neurotransmitter levels through the multidirectional mechanisms of the gut–brain axis and the influence on the composition of bacterial species. As a consequence of the metabolic changes induced by such regulation, a modification in the amounts of neurotransmitters such as glutamate and gamma-aminobutyric acid (GABA) is observed. GABA is the neurotransmitter that inhibits overexcitation of the nervous system, while glutamate is the most important excitatory neurotransmitter. Many species of gut bacteria, including *Lactobacillus*, are capable of producing GABA, while other strains affect glutamate and serotonin metabolism [66].

A key metabolic aspect of the KD is its effect on the GABA/glutamate ratio in the brain, where βHB, through inactivation of glutamate dehydrogenase, redirects glutamate metabolism from a catabolic process to the GABA biosynthesis pathway via glutamic acid decarboxylase [65]. βHB not only provides an alternative energy source for neurons with impaired glucose utilization, but also reduces glutamate neurotoxicity by improving mitochondrial metabolism and reducing oxidative stress [68].

### 3.5. Controversies Surrounding the KD in the Context of Selected Organs

Implementing the KD in patients with diverse diseases presents numerous challenges. The diet requires strict adherence to specific macronutrient ratios, which can be demanding in clinical practice and often leads to reduced long-term compliance. Continuous medical supervision and regular monitoring of biochemical parameters are therefore essential to ensure both efficacy and safety [69].

Long-term KD use is also associated with several controversies, primarily due to the scarcity of studies exceeding 5–6 years and uncertainty about the safety of maintaining such a restrictive diet over extended periods. Limiting the intake of fruits, vegetables, and whole grains can lead to deficiencies in vitamins (A, E, B6, folate) and minerals (magnesium, calcium, potassium), as well as insufficient fiber consumption, which may promote acid–base imbalance and impair gastrointestinal function [70,71]. Additionally, prolonged adherence has been linked to lipid metabolism disturbances, including elevated triglycerides and LDL-C, while the effects on cardiovascular risk remain inconclusive [71,72,73]. Some studies have reported increases in inflammatory markers such as CRP, potentially favoring the development of atherosclerosis. The risk of nephrolithiasis is also significant, reaching up to 25% in children after six years of KD use and approximately 7.9% in adults [70]. Meta-analyses covering shorter periods (6–24 months), however, suggest that low-carbohydrate diets do not significantly impair renal function in adults [74].

Adherence remains a major limitation, as most patients discontinue the KD within months. While short-term use (several weeks up to one year) can provide meaningful clinical benefits, prolonged implementation (≥2 years) is often associated with increased risk of lipid abnormalities without clear advantages over less restrictive dietary approaches [75]. In a large study of 491 adults, García-Gorrita et al. (2025) confirmed the effectiveness of the KD in reducing fat mass, yet participant retention declined markedly over nine months, with only 41 individuals completing the program [76].

## 4. Analytical Challenges and High-Throughput Metabolomic Approaches in KD Research

### 4.1. Analytical Challenges in KD Metabolomics

Over the last few years, the main analytical methods used in metabolomics have undergone significant development. One of the earliest and still widely applied techniques in KD-related research is NMR spectroscopy. This method has been employed for more than 40 years in metabolic studies, profiling of body fluids, and tissue analysis due to its ability to characterize the chemical composition of complex mixtures. However, its role has been partly superseded by more advanced technologies, such as mass spectrometry (MS) and two-dimensional MS, which provide higher sensitivity, broader compound coverage, and compatibility with various separation techniques [7,77]. The key advantage of MS-based methods lies in their ability to simultaneously identify and quantify numerous compounds in complex biological matrices, making them indispensable tools in modern metabolomics [8,45].

Currently, chromatographic methods coupled with mass spectrometry are most commonly used, with LC-MS and GC-MS combinations selected depending on the specific requirements of a study. These approaches enable both qualitative and quantitative determination of hundreds of endogenous compounds in body fluids and tissues. The challenge, however, is to capture metabolites with highly diverse physical and chemical properties. This can be addressed through the careful selection of chromatographic columns, detectors, solvents, mobile phases, analysis parameters, and sample preparation methods, thereby increasing the number of metabolites identified in a single run from hundreds to thousands. Table 2 and Table 3 present examples of the use of LC and GC in KD-related metabolomics, highlighting the diversity of compounds detected with these approaches.

In addition to MS-based techniques, other bioanalytical assays continue to play a supportive role in metabolomics. These include kinetic and enzymatic colorimetric tests (e.g., for urea, glucose, cholesterol, triglycerides, and lipoproteins), immunological electrochemiluminescence assays (for hormones such as cortisol, insulin, and C-peptide) [78], enzyme-linked immunosorbent assays (ELISAs), and chemiluminescent methods for vitamin D quantification [79]. Electrospray ionization mass spectrometry (ESI-MS) has also been applied in specific contexts [80]. Nonetheless, chromatographic and MS-based platforms clearly surpass these tests in scope, sensitivity, and the breadth of analytes covered. Equally important is the integration of advanced statistical approaches with publicly available databases, such as the Human Metabolome Database, which substantially enhances data interpretation and biological contextualization [5,11,78,79,80,81,82,83,84,85].

### 4.2. High-Throughput Metabolomic and Imaging Approaches

While analytical barriers exist, recent high-throughput studies have demonstrated how these methods directly contributed to uncovering KD mechanisms. Over the past decade, targeted and untargeted LC-MS/GC-MS studies have mapped KD-driven pathway remodeling in humans and patients with epilepsy, firmly anchoring clinical effects to biochemistry. In healthy adults, a three-week KD consumed without caloric restriction produced a marked shift toward mitochondrial fatty acid oxidation with elevations in free fatty acids and acylcarnitines, a redistribution of serum amino acids (reduced glucogenic amino acids, higher BCAA), and changes in tryptophan–kynurenine metabolism consistent with anti-inflammatory signaling—metabolic features that co-occurred with lower insulin and triglycerides [78]. In drug-resistant epilepsy, a short-term KD (2–4 weeks) increased circulating ketone bodies, diverse lipids, and catabolites upstream of acetyl-CoA/propionyl-CoA—including branched-chain amino acid breakdown products and GABA analogs—implicating enhanced anaplerosis and neurotransmitter precursor flux as candidate antiseizure mechanisms [86]. Preclinical LC-MS work complements these human data: hippocampal and plasma metabolomics in KD-fed rats demonstrated a coordinated downshift of kynurenine-pathway intermediates, linking KD to reduced neurotoxic tryptophan catabolites and plausible immuno-excitatory modulation [87]. The scientific literature provides evidence for the existence of metabolic disorders accompanying epilepsy, particularly in choline metabolism. Lalwani and colleagues conducted studies on brain tissue collected after the death of patients with epilepsy, using nuclear magnetic resonance spectroscopy (^1^H-NMR) and direct injection liquid chromatography coupled with tandem mass spectrometry (DI/LC-MS/MS). Analysis of frontal cortex samples revealed changes in the concentration of O-acetylcholine and adenosine monophosphate (AMP), indicating disturbances in choline metabolism and the β -oxidation of fatty acids [88]. Together, these MS-based studies move beyond descriptive ketosis to pinpoint specific nodes (fatty-acid β-oxidation, BCAA and kynurenine metabolism) that plausibly mediate seizure control, metabolic benefits, and inflammation resolution.

NMR-based metabolomics has extended this system’s view into the central nervous system by simultaneously profiling serum and cerebrospinal fluid (CSF) during controlled interventions. In a cross-over trial of individuals at risk for Alzheimer’s disease, a modified Mediterranean ketogenic diet (MMKD) selectively altered CSF amino acids and increased CSF BCAA-degradation products while reprogramming serum lipoprotein composition and lowering systemic inflammatory signatures; cross-compartment correlation networks further showed coordinated shifts between serum ketone bodies and CSF metabolites, supporting a diet-to-brain metabolic relay [89]. These findings indicate that ketogenic dietary patterns can rewire central BCAA/TCA-adjacent flux and lipid handling in ways that intersect with neurodegenerative risk biology.

In vivo MRS/MRI studies provide mechanistic, tissue-resolved readouts that align with the biochemical profiles above. Longitudinal 1H-MRS in adults with intractable epilepsy documented pronounced increases in absolute brain GSH—particularly in gray matter—alongside ~50% seizure reduction during KD with emulsified MCT oil, strengthening the case that KD augments neuronal redox capacity as part of its anticonvulsant action [62]. Independently, cross-sectional MRS in KD-treated patients reported higher cerebral GSH levels versus controls [63]. In normal rats maintained on a four-month KD, combined structural MRI, spectroscopy, and tractography revealed increased brain glutamine, glutamate, GSH, and N-acetylaspartate together with reconfigured connectivity in striatum and hippocampal formation—evidence for durable neurobiological adaptations of glutamatergic/redox metabolism and white matter architecture under sustained ketosis [64]. These imaging results substantiate metabolomic inferences by demonstrating redox-and neurotransmission-related changes directly in brain tissue and by mapping circuit-level plasticity under KD [42].

Stable isotope tracing has been pivotal for quantifying pathway fluxes that static metabolite levels cannot resolve. Dual-tracer (C^13^-BHB and C^13^-AcAc) approaches with LC-MS/MS readout and metabolic flux analysis have clarified ketone turnover kinetics and the rapid interconversion between acetoacetate and βHB in vivo, addressing long-standing biases of single-tracer methods and enabling rigorous estimation of hepatic production versus peripheral disposal during ketosis. Coupled with C^13^ tracing into TCA intermediates and neurotransmitter pools, these strategies allow quantification of KD-induced shifts in carbon routing (e.g., enhanced use of ketone-derived acetyl-CoA for oxidative metabolism) and, in seizure contexts, accelerated glutamate–glutamine cycling, thereby connecting ketone availability to neuronal energy and transmitter homeostasis [90].

Miller and colleagues conducted a 12-week intervention in 29 active adults using targeted analyses of mitochondrial function in skeletal muscle biopsies and blood serum. The ketogenic diet significantly improved metabolic parameters, increasing fat oxidation at rest and reducing insulinemia. Mitochondrial analyses showed improved efficiency in the utilization of fatty acid substrates, with minimal utilization of ketone substrates and a significant increase in muscle triglycerides. The study aimed to understand the mechanisms of action of the ketogenic diet in combination with physical training [56].

In animal model studies, Mayengbam and colleagues performed an untargeted metabolomic analysis using LC-QTOF-MS on brain tissue from young mice of three strains fed a ketogenic or standard diet. The analysis revealed significant changes in the brain’s metabolic profile, including a decrease in methionine and carnitine concentrations, an increase in glutathione, pantothenate, and glutamine concentrations, and changes in metabolites of nucleotide, bile acid, and folate pathways. The aim was to understand the mechanisms of action of the ketogenic diet on brain structure development [91].

Castro and his team used targeted metabolomics with the MxP Quant 500 platform in ApoE^−^/^−^ mice fed a ketogenic diet for 12 weeks. In the KD group, there was an increase in the levels of amino acids that are precursors of GSH, bile acids, and methylation metabolites, as well as a decrease in the levels of phosphatidylcholine, sphingolipids, cholesterol esters, and ceramides. The aim was to elucidate the mechanisms of action of the ketogenic diet in the context of atherosclerosis progression [92].

Finally, integrated microbiome–metabolome studies have started to bridge dietary composition, host metabolism, and clinical response. In children with drug-resistant epilepsy, three months of KD produced broad serum metabolomic remodeling; importantly, specific lipids (plasmalogens) correlated with seizure reduction and with beneficial gut microbes, while potentially unfavorable taxa showed opposite associations—suggesting that metabolite–microbe networks may modulate, or even predict, therapeutic response to the KD [93]. Such datasets support the view that high-throughput omics can yield predictive signatures for personalization rather than merely describing ketosis [94].

Collectively, these examples demonstrate that high-throughput platforms are not ancillary, but mechanistically decisive: LC-MS/GC-MS delineate pathway targets of the KD (fatty-acid oxidation, BCAA, and kynurenine metabolism), NMR metabolomics exposes coordinated blood–CSF remodeling, in vivo MRS/MRI verifies redox and neurotransmission changes within brain tissue and maps structural adaptations, and C^13^ tracing quantifies carbon-flux reprogramming. This multimodal evidence base substantiates the conclusion that metabolomics and complementary high-throughput approaches are essential for explaining, monitoring, and ultimately individualizing KD therapy.

Table 2 and Table 3 present examples of the use of liquid and gas chromatography in metabolomic research, taking into account the metabolites determined by the method.

### 4.3. Clinical and Safety Considerations

Implementing the KD across diverse patient populations presents significant challenges related to adherence, tolerability, and long-term safety. Short-term benefits are often accompanied by transient side effects, such as fatigue, constipation, gastrointestinal discomfort, and electrolyte disturbances, which can be particularly burdensome in older patients or those with comorbidities [70,71]. These symptoms typically resolve with medical supervision and dietary adjustments, but highlight the importance of structured monitoring during the initiation phase.

Long-term use of the KD raises more complex concerns. Restrictive macronutrient ratios and the exclusion of fruits, vegetables, and whole grains can contribute to deficiencies in vitamins (A, E, B6, folate) and minerals (magnesium, calcium, potassium), along with insufficient dietary fiber [70,71]. Such imbalances may promote gastrointestinal dysfunction, acid–base disturbances, and increased cardiovascular risk. Clinical and experimental evidence also suggests potential hepatic and renal complications: rodent studies have demonstrated inflammatory and vascular changes in the liver after prolonged KD exposure [95], while in humans, elevated nitrogen excretion and hyperfiltration have been linked to an increased risk of nephrolithiasis [96]. Indeed, kidney stones occur in up to 25% of children after six years of KD use and in approximately 8% of adults [70], whereas reductions in bone mineral density and increased fracture risk have been reported after long-term treatment [97]. In women of reproductive age, disruptions of the one-carbon pathway and DNA methylation under KD may have developmental consequences for offspring [98], while in children, prolonged adherence has been associated with growth retardation [97]. Elderly patients are also at risk of malnutrition, sarcopenia, and water–electrolyte imbalance [99], further limiting the feasibility of sustained KD application.

Taken together, these observations underscore that the safety and efficacy of the KD are highly individualized and dependent on patient characteristics, diet composition, and intervention duration. Continuous clinical supervision with regular monitoring of biochemical and nutritional status is therefore essential [100]. Importantly, metabolomics and related omics platforms provide a unique opportunity to identify early biomarkers of adverse effects, thereby enabling more precise risk stratification and safer, personalized implementation of the KD in chronic therapy [94].

## 5. Potential of the KD in the Treatment of Various Diseases and Disorders

The spectrum of therapeutic use of the KD covers a wide range of conditions. The KD has long been recognized as an extremely effective dietary approach for the treatment of drug-resistant epilepsy and has increasingly attracted researchers’ attention over the past decade for a variety of diseases beyond epilepsy, from obesity to malignancies [101]. For neurodegenerative diseases, emerging evidence links the KD to improved cognitive function, reduced motor symptoms, and increased mitochondrial activity in patients. It shows promising potential in mitigating disease progression by affecting metabolic processes and providing neuroprotective benefits by inducing ketosis and leading to the production of ketone bodies, such as βHB, which reduce oxidative stress and modulate inflammatory pathways [102]. The diet also shows clinical impact in the treatment of Alzheimer’s disease (AD) and Parkinson’s disease (PD) [103]. The efficacy and safety of the KD are also currently being evaluated in studies on its potential benefits for the gut–brain axis [104]. In the context of metabolic diseases, the KD helps lower glycated hemoglobin levels, reduces insulin requirements, and promotes weight and visceral fat reduction in diabetes and obesity. Positive effects are also observed in heart disease, where the diet improves the lipid profile by lowering LDL, raising HDL, and lowering triglycerides and blood pressure [61,67]. In the area of mental health, preliminary studies suggest improvement in mood and quality of life in illnesses such as depression, bipolar disorder, and schizophrenia [105]. Therefore, as can be seen, the KD presents itself as a good candidate for complementary therapy, although its specific use depends on the type and severity of the disease. The significance of nutrition as an important aspect of disease treatment strategies is increasingly emphasized. Also, there is a need for further research to refine the therapeutic applications of the KD and to better understand its potential long-term effects [103].

### 5.1. Drug-Resistant Epilepsy

For years, significant improvements have been observed in children with drug-resistant epilepsy following diets designed to put the patient into a state of ketosis. In a 2022 study [28], the success rate of the therapy, defined as no seizures or a 50% reduction in seizure frequency, reached 63% among a group of adult subjects and almost 76% among a group of child subjects. In subjects who were still experiencing episodes, a reduction in seizure severity of 68% in adults and almost 64% in children was observed.

What is problematic in studies on the efficacy of the KD is that some patients do not maintain the dietary regime throughout the follow-up period. This affects the results by reducing the study group and missing long-term results, including side effects. Modifications that are more patient-friendly and have similarly effective anticonvulsant effects may be the answer. An example is the MAD. The results of studies on the efficacy of this therapy are inconclusive. There are studies that show little difference in the effect of the cited diets, but there are also publications that discuss the superiority of the classical KD. However, even if one observes the superiority of the KD over the MAD in terms of reducing and alleviating epileptic seizures, it is a minor difference. Given the greater flexibility and easier-to-follow principles, there is some advantage of the MAD over the KD [26,62].

**Figure 5:**
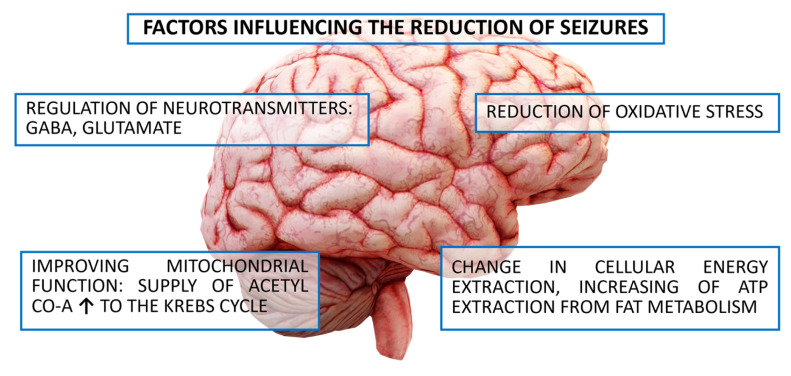
Factors influencing the reduction in epileptic seizures following a KD. ↑—indicates an increase in acetyl Co-A.

The KD was primarily developed for the treatment of children suffering from drug-resistant epilepsy. The exact mechanism has not been determined. The antiepileptic effect of ketogenic therapy is pleiotropic and includes several levels that determine its anticonvulsant and anti-neurodegenerative effects. These include, first and foremost, a change in the acquisition of energy from glucose to ketone bodies. The conversion of fats to free fatty acids allows hepatic β-oxidation to produce ketone bodies (acetone, AcAc and βHB) actively involved in the Krebs cycle, which determines ATP production. There is a change in cellular energy acquisition with a doubling of ATP production when ketones are on the substrate side, followed by a reduction in oxidative stress, regulation of the neurotransmitters GABA and glutamate, and an improvement in mitochondrial function through the supply of substrates (acetyl-CoA) to the Krebs cycle. This energetically beneficial conversion in combination with chronic ketosis appears to directly and indirectly modulate neuronal and neuroglia function, which is responsible for key neuroimmune processes in epilepsy. The result is a neuroprotective effect of the KD with promotion of inhibitory (GABAergic) transmission and inhibition of excitatory (NMDA-dependent N-methyl-D-aspartate) neurotransmission. Furthermore, a favorable energy balance stabilizes the synaptic system, which plays a key role in epileptic seizure propagation [30,106,107]. Figure 5 presents the main factors influencing the reduction of seizures.

### 5.2. Obesity, Type 2 Diabetes, and Insulin Resistance

The KD shows significant benefits in controlling blood glucose levels, HbA1c, and insulin sensitivity in patients with type 2 diabetes [101]. Carbohydrate restriction, particularly through VLCKD, leads to better glycemic control compared to low-carbohydrate diets, as confirmed by numerous clinical studies [108]. These positive effects may contribute to such improved glycemic control that it even leads to diabetes remission [67]. Specifically, VLCKD leads to significant improvements in glycemic indices, including fasting blood glucose, with an average decrease of approximately 8.85 mg/dL, and HbA1c, with an average decrease of 0.43% [109]. The therapeutic effects are relatively rapid—already 24 weeks after the implementation of VLCKD, patients achieved HbA1c levels in line with standards of around 6.2%. Long-term follow-up is equally promising—in a two-year study, up to 53.5% of patients achieved remission of type 2 diabetes, while diabetes reversal occurred in more than 50% of patients and complete remission in almost 8% [110]. The practical benefits of this dietary intervention are particularly evident in the context of pharmacotherapy, where patients with type 2 diabetes on a KD often reduce or completely discontinue the intake of insulin and other antidiabetic drugs. The mechanisms responsible for these beneficial effects are closely related to the effects of the KD on insulin resistance and glucose metabolism. The KD improves cellular sensitivity to insulin and reduces hyperglycemia through fundamental changes in energy metabolism. These metabolic effects are manifested by lower fasting insulin levels and improved tissue insulin sensitivity, which is particularly evident in patients with insulin resistance [67,101]. Clinical evidence of these benefits comes from studies in which patients with hyperinsulinemia reported significant improvements in HDL levels and decreases in triglycerides and body weight, as well as a reduction in insulin resistance through reduced insulin levels and homeostatic model assessment of insulin resistance (HOMA-IR) [61]. Furthermore, the reduction of inflammation and oxidative stress due to ketones, especially βHB, improves pancreatic β-cell function [111]. These metabolic benefits translate directly into efficacy in weight management, where the KD is effective in promoting weight loss and improving metabolic markers in obese individuals. Comparative studies consistently show that the KD, both short- and long-term, helps to reduce weight, BMI, waist circumference, and blood pressure and also improves insulin resistance, with the KD found to be more effective than low-fat diets in numerous studies, both short- and long-term [67,101]. The actual clinical results are impressive: VLCKD resulted in significant weight loss with an average weight reduction from 7.5 kg after 1 month to 21.5 kg after 12 months, with a BMI reduction from 3.25 after 1 month to 7.11 after 1 year. Also of note, the reduction in fat mass was approximately 11 kg, which was clearly greater than in the control groups, while the reduction in waist circumference averaged 16.5 cm, which was also greater than with other diets. It is clinically important that the KD effectively reduces visceral fat, which is strongly associated with metabolic risk, while maintaining lean body mass despite significant overall weight loss [67,109]. These observations are corroborated in meta-analytical analyses, which showed that the KD was significantly more effective than other diets in reducing body weight with an standardized mean difference (SMD) of −5.63 (*p* = 0.008) and reducing waist circumference with an SMD of −2.32 (*p* = 0.04) [112].

The biochemical mechanisms responsible for these benefits are complex and multifaceted. Mechanisms of benefit of the KD include increased satiety, decreased lipogenesis, and increased fat oxidation. A key element is that the production of ketone bodies, particularly βHB, act to suppress hunger and cravings, making it easier to maintain a caloric deficit. On a metabolic level, the KD acts by lowering insulin levels, increasing fat oxidation, reducing lipogenesis, and potentially inducing epigenetic changes that protect against oxidative stress and inflammation [67,101].

In the context of lipid profile, the KD shows beneficial effects, having a better impact on lipid profiles compared to low-fat diets. Meta-analyses show a significant decrease in triglycerides with an SMD of −0.36 (*p* = 0.0001) and an increase in HDL with an SMD decline of 0.28 (*p* = 0.003), with no significant changes in LDL and total cholesterol, suggesting relative cardiovascular safety [112]. Despite the promising therapeutic benefits, the implementation of the KD requires caution and appropriate medical supervision. Caution should be exercised when combining the KD with drugs such as sodium–glucose cotransporter 2 inhibitors or insulin, due to the risk of hypoglycemia or ketoacidosis. Nevertheless, VLCKD has been evaluated as a safe and well tolerated method provided it is carried out under specialist supervision, with side effects such as headache, constipation, and fatigue usually being mild and transient [109,112]. An important aspect to consider in clinical practice is that the metabolic benefits attributed to the KD are strongly dependent on weight loss rather than ketogenesis itself. This means that weight loss is a key factor in improving insulin sensitivity and glycemic control in people with type 2 diabetes and obesity [113]. Additionally, due to the strictness and restrictiveness, many patients find it difficult to adhere to the KD in the long term, highlighting the need for a personalized approach and support in the implementation of this dietary intervention [111].

### 5.3. Neurodegenerative Diseases (Parkinson’s and Alzheimer’s Disease)

The KD is attracting increasing interest as a potential therapeutic support in neurodegenerative diseases, particularly Parkinson’s disease (PD) and Alzheimer’s disease (AD). A diet based on high fat intake and carbohydrate restriction leads to the production of ketone bodies, which can provide an alternative source of energy for the brain, bypassing the impaired glucose metabolism characteristic of neurodegenerative diseases [114,115].

**Figure 6:**
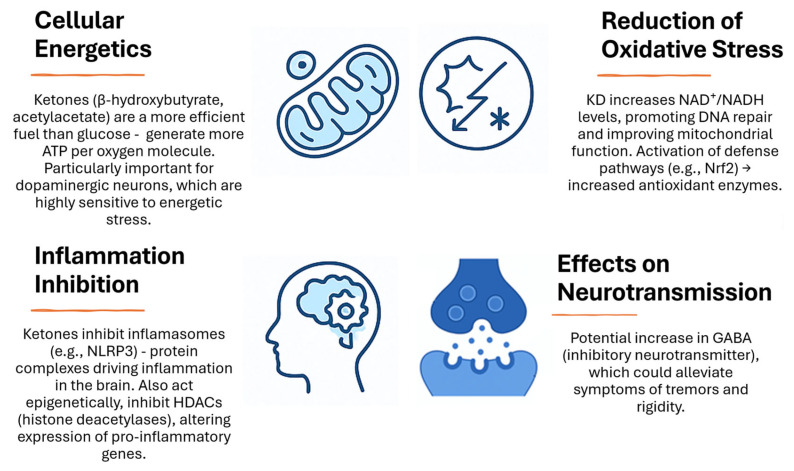
Mechanisms of action at the cellular level in neurodegenerative diseases (PD and AD).

The basis of the action of the KD is the metabolic switch from glucose to ketones as the main source of energy for the brain. Ketone bodies, mainly βHB and acetoacetate, can cross the blood–brain barrier and provide alternative fuel for neurons. This mechanism takes on particular importance given the impaired glucose metabolism observed in neurodegenerative diseases. Schematically, the mechanism of KD action at the cellular level in neurodegenerative diseases is presented in Figure 6. Ketosis exerts neuroprotective effects through several pathways. It improves mitochondrial function, increasing the efficiency of ATP production and reducing oxidative stress. Ketone bodies exhibit anti-inflammatory properties, which may slow neurodegenerative processes. In addition, the KD may influence the stabilization of neuronal membranes and improve signaling between nerve cells [30,34,116,117,118,119].

The positive effects of the KD on neurodegenerative diseases such as PD and AD have been confirmed by numerous studies. In PD, there is a progressive loss of dopaminergic neurons in the black matter, leading to characteristic motor symptoms. Preclinical studies suggest that a KD may protect dopaminergic neurons by improving mitochondrial function and reducing oxidative stress. Mitochondrial deficits and increased oxidative stress are also observed in PD, which may be partially mitigated by ketone metabolism [120]. Dopaminergic neurons in the black matter are particularly sensitive to energy deficiency due to their high metabolic demand and low mitochondrial reserve [121]. Ketone bodies, unlike glucose, generate more ATP per unit of oxygen, which can compensate for energy deficits in PD [122]. They also improve cellular bioenergetics and reduce the production of reactive oxygen species [123]. Studies in animal models have shown that βHB increases ATP levels in the brain and improves mitochondrial function, resulting in better neuronal survival [124]. The study by Phillips et al. (2018) involved 47 PD patients who followed either KD or a low-fat diet for 8 weeks [125]. Both groups showed improvement on the Movement Disorder Society-Unified Parkinson’s Disease Rating Scale (MDS-UPDRS), but the ketogenic group showed greater improvement in non-motor symptoms such as fatigue, daytime sleepiness, pain, urinary problems, and cognitive function. No significant differences in motor symptoms were observed between the groups. It should be noted that adverse effects of KD use were also observed, which included intermittent exacerbation of tremor and/or rigidity [125]. Yudkoff et al. (2007) observed that the KD may increase levels of GABA, an inhibitory neurotransmitter that potentially alleviates tremors and stiffness [126]. A clinical pilot study [127] of patients with PD who followed a KD for 4 weeks showed improvement in motor symptoms (UPDRS-III scale). In 2022, Tidman published a case study of a 68-year-old woman with PD who had followed a KD for 24 weeks. Improvements were observed in health biomarkers (including HbA1c, CRP, triglycerides, fasting insulin), weight loss, and anxiety symptoms, while improvements in depression were minimal [128]. In another study, in which PD patients followed the KD diet for 24 weeks, improvements in cognitive function, mood, motor and non-motor symptoms, and reductions in pain and anxiety were observed. This dietary intervention proved to be an effective tool in alleviating the symptoms of this disorder while improving patients’ quality of life [129]. Studies conducted on animal models have shown that the KD may reduce α-synuclein aggregation and neuroinflammation [130] and reduce damage to the mitochondrial respiratory chain. The KD protected dopaminergic neurons from degeneration in the substantia nigra. A reduction in oxidative stress, normalization of glutathione levels, and increased activity of antioxidant enzymes (e.g., SOD, catalase) were observed. Ketones (especially βHB) had a neuroprotective effect, limiting inflammatory processes and mitochondrial stress [131]. Current experimental evidence and preliminary clinical data suggest that the KD may be a promising strategy for supporting PD therapy, but further randomized studies with long-term follow-up are needed.

AD is characterized by progressive neurodegeneration associated with, among other things, β-amyloid (Aβ) accumulation, tau protein hyperphosphorylation, mitochondrial dysfunction, and chronic brain inflammation. The KD, which induces the production of ketone bodies, is being investigated as a potential metabolic intervention that may modify the course of the disease by improving neuronal function, reducing oxidative stress, and inhibiting neuroinflammatory processes [132]. AD is also characterized by impaired glucose metabolism in the brain, which some researchers refer to as “type 3 diabetes” [133]. In this context, an alternative energy source in the form of ketones may be particularly beneficial [134]. Ketone bodies can be an alternative source of energy for neurons, potentially compensating for the energy deficits characteristic of AD. In this disease, there is a reduction in glucose utilization in the brain, leading to a condition known as “brain energy starvation” [135]. Ketones, in particular βHB, are able to cross the blood–brain barrier and provide an alternative source of energy, compensating for glucose deficits [29,132]. Ketone bodies may also improve cognitive function by providing energy support to neurons and reducing Aβ accumulation, as observed in transgenic mice modeling AD [136,137]. In animal models, the KD was associated with improvements in metabolic, behavioral, and neuropathological parameters. For example, a 43-day KD intervention in mice led to a significant reduction in amyloid β40 and β42 deposits [137]. In the 3×Tg-AD model, the KD improved cognitive function and enzymatic activity in the citric acid cycle [138]. Phillips and colleagues conducted a randomized controlled trial involving 26 patients with AD. During 12 weeks of KD use, a significant improvement in daily functioning (according to the AD Cooperative Study–Activities of Daily Living (ADCS-ADL) scale) was observed: +3.13 ± 5.01 points, *p* = 0.0067, as well as quality of life (according to the Quality of Life in AD (QOL-AD) questionnaire): +3.37 ± 6.86 points, *p* = 0.023 scores. Changes in cognitive function (Addenbrookes Cognitive Examination–III scale (ACE-III)) did not reach statistical significance (*p* = 0.24). Patients on KD showed improvement in daily functioning and quality of life, as well as greater beneficial changes in cardiovascular risk factors, and adverse effects were mild [139]. In another study, Henderson et al. (2009) conducted a 3-month pilot study in which 11 patients with early AD received the KD supplemented with MCT [140]. Improvements in cognitive function were observed, particularly in patients in the early stages of the disease and without the presence of the APOE4 allele. An average improvement of 4.1 points was achieved in the ADAS-Cog test, but the effects subsided after a 4-week break [140]. Studies have shown that AD is associated with impaired glucose utilization in the brain, particularly in cortical areas related to memory. Ketone bodies provide an alternative source of energy, bypassing deficits in glucose metabolism. Ketones such as βHB are efficiently transported to the brain by monocarboxylate transporter 1 (MCT1) and can provide up to 70% of energy under conditions of ketosis [141]. Current evidence suggests that the KD may alleviate key pathologies of AD, but its long-term efficacy requires further study. The KD shows promise as an alternative therapeutic tool in neurodegenerative diseases such as PD and AD, offering neuroprotective benefits with fewer dietary restrictions. The development of these strategies may make ketone therapy more accessible to a wider group of patients. Although the mechanisms of action are biologically sound and preliminary clinical trial results are encouraging, further research is needed to fully determine its place in the treatment of PD and AD. Recent metabolomic profiling of Alzheimer’s disease and Parkinson’s disease has revealed alterations in lipid metabolism, neurotransmitter precursors, and oxidative stress markers under KD, supporting its potential neuroprotective effects [87,89].

### 5.4. Cancers

Numerous studies are being conducted on various types of cancer and the impact of the KD on their treatment. The use of the KD in cancer patients is based on the Warburg effect, which means that many cancer cells have an increased demand for glucose and a limited ability to use ketone bodies as an energy source. As a result of the lack of readily available glucose, the main energy substrate, cancer cells undergo apoptosis as a consequence of starvation [35]. In addition, it has been observed that an increase in the level of ketone bodies shows anticancer effects through gene expression modification, oxidative stress induction, and inhibition of proliferative pathways [142].

Mitochondrial dysfunction can lead to increased expression of certain oncogenes associated with the signaling pathway, which plays a key role in regulating cancer cell metabolism. Activation of this pathway, also associated with insulin signaling, promotes metabolic changes that favor cancer cell survival, including increased resistance to apoptosis, decreased β-oxidation of fatty acids, and increased lipogenesis in the cytosol. Therefore, the hypothesis that a very low-carbohydrate diet may limit the progression of certain cancers seems reasonable, although current data remain at the stage of preliminary observations [143,144,145].

In preclinical models of pancreatic cancer in mice, the KD caused a decrease in glucose and insulin levels and reduced phosphoinositide 3-kinaze (PI3K)/protein kinase B (Akt)/mechanistic target of rapamycin (mTOR) activation in pancreatic islet cells, which limited the proliferation and development of neuroendocrine tumors [146]. The KD also shows potential as a strategy to support cancer therapy, especially in synergy with other treatment methods such as chemotherapy. However, at present, there is no clear clinical evidence confirming its effectiveness in large randomized studies. In models of neuroblastoma and breast cancer, the use of the KD in combination with drugs (e.g., chemotherapy, PI3K inhibitors) increased the effectiveness of treatment, among other things by inhibiting angiogenesis and inducing oxidative stress [147]. Ketones such as βHB can alter the redox balance by reducing nicotinamide adenine dinucleotide phosphate reduced form (NADPH) and glutathione in pancreatic ductal adenocarcinoma (PDAC) cells, making them more susceptible to chemotherapy. In a study conducted by Yang et al. (2022), it was observed that chemotherapy alone and the KD combined with chemotherapy increased the NADH/NAD ratio in tumors [148]. Furthermore, tumors with the highest NADH/NAD values showed a regression of lesions. The use of the diet in combination with cytostatic treatment also led to a decrease in the concentration of glutathione and NADPH in the tumor tissue—substances responsible for neutralizing ROS—and, at the same time, to an increase in lipid oxidation, which is an additional indicator of oxidative stress. The results indicate that the KD leads to an increase in the systemic level of βHB, which is used by pancreatic ductal adenocarcinoma under conditions of limited glucose availability. The metabolism of βHB results in the accumulation of intracellular NADH, which cannot be effectively balanced by systemic redox mechanisms based on the 3-hydroxybutyrate/AcAc system. This metabolic state sensitizes ductal PDAC tumor cells to chemotherapy, which further enhances NADH accumulation, ultimately leading to tumor cell death and tumor shrinkage [148,149].

It should also be noted that there is evidence that the KD may enhance the immune response against tumors in the context of immunomodulatory therapy. In their study, Ferrere et al. (2021) demonstrated that both KD and oral administration of βHB restored the efficacy of antibody against programmed cell death protein 1 (anti-PD-1) therapy in mice in which PD-1 blockade alone was ineffective [150]. Mechanistically, 3-hydroxybutyrate prevented PD L1 induction in myeloid cells while promoting the expansion of CXCR3-marked T cells, resulting in T cell-dependent tumor growth inhibition [150]. Metabolomic profiling conducted by Wang and colleagues in 2024 revealed a statistically significant reduction (*p* < 0.05) in AcAc concentrations within radioiodine-resistant differentiated thyroid carcinoma (RAIR-DTC) specimens compared to conventional DTC cases. Experimental evidence suggests that elevating AcAc through KD interventions promotes enhanced expression of the sodium iodide symporter (NIS) and thyroid-stimulating hormone receptor (TSHR), resulting in substantially improved radioiodine incorporation within papillary thyroid carcinoma cells. Preclinical studies revealed that AcAc elevation coincided with suppressed tumor proliferation, diminished Ki-67 markers, and enhanced apoptosis. These findings suggest that ketogenic dietary protocols may provide a complementary therapeutic approach when combined with radioiodine treatment, enhancing both cellular radiotracer uptake and cytotoxic efficacy against radioiodine-refractory thyroid malignancies [35].

By “starving” cancer cells of glucose and reducing the direct effects of insulin on cell growth, the KD appears to be promising as an aid in certain types of cancer therapy and deserves further and more in-depth research. The KD may be a promising strategy to support cancer treatment, especially in combination with chemotherapy and radiotherapy. Its use should be considered on an individual basis, taking into account the type of cancer, the stage of the disease, and the patient’s ability to adhere to a restrictive dietary regimen. Further well-designed randomized trials are needed to confirm its efficacy and safety.

Implementing the KD in a population of patients with various diseases presents numerous challenges. The diet requires strict adherence to macronutrient ratios, which can be challenging. The long-term safety of the KD in patients has not been fully documented. There are concerns about the diet’s impact on the cardiovascular system, kidney function, and bone density. Continuous medical supervision and regular monitoring of patients’ biochemical parameters are necessary [69].

### 5.5. Ketogenic Therapy in Developmental Neurological Disorders

The development of neurogenetic diagnostics with increasingly frequent molecular diagnosis enables personalized treatment, including ketogenic therapy. Genetic diagnosis has a strong influence on the efficacy and safety of the KD, and absolute indications for its implementation are glucose transporter type 1 deficiency syndrome (GLUT1DS) and pyruvate dehydrogenase complex deficiency (PDCD). Preferred additional applications of the KD include genetic epilepsies and associated developmental and epileptic encephalopathies (DEE), such as Dravet syndrome, associated with a pathogenic variant of the SCN1A gene (sodium voltage-gated channel alpha subunit 1), as well as other sodium channelopathies (SCN2A, SCN8A) or potassium channelopathies (KCNQ2). Increasing clinical data indicate the effectiveness of ketogenic therapy in other severe DEEs, such as cyclin-dependent kinase-like 5 (CDKL5) deficiency disorder, DEE-STXBP1 (syntaxin-binding protein 1), PCDH19-related epilepsy (protocadherin 19), and GRIN2A-related epilepsy (glutamate receptor, ionotropic, N-methyl D-aspartate 2A).

**Figure 7:**
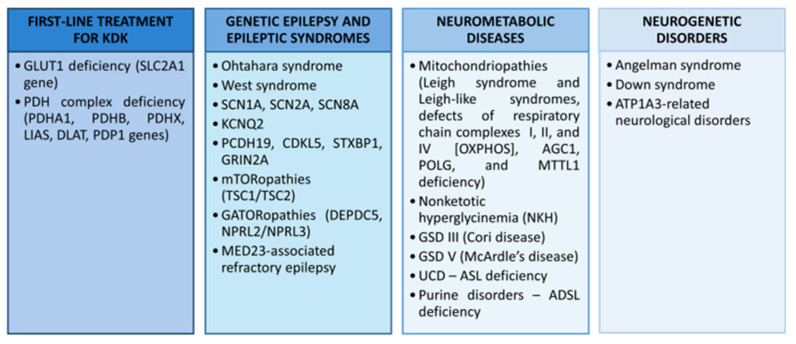
Application of ketogenic therapy in neurological diseases [31,102,151,152,153].

The high effectiveness of the KD is also observed in mTOR pathway disorders represented by tuberous sclerosis, associated with pathogenic variants in the TSC1/TSC2 (tuberous sclerosis complex) genes, and GATORopathies, dependent on pathogenic variants in the DEPDC5, NPRL2, and NPRL3 genes. The list of other neurometabolic disorders in which the inclusion of the KD may bring the desired effect and which for years were considered contraindicatory to the inclusion of the KD is also systematically expanding. These include, in particular, energy disorders from the group of mitochondriopathies (Leigh syndrome and Leigh-like syndromes, defects of the OXPHOS respiratory chain complex), neurotransmission disorders (NKH—non-ketotic hyperglycinemia), and glycogen storage disorders GSD III and V (Glycogen storage disease). In recent years, argininosuccinate acidemia has been associated with argininosuccinate lyase (ASL) deficiency, which belongs to urea cycle disorders (UCDs) and adenylosuccinate lyase (ADSL) deficiency, representing purine metabolism disorders. The KD is also highly effective in classic neurogenetic syndromes associated with a high incidence of drug-resistant epilepsy, such as Down syndrome (trisomy 21) and Angelman syndrome, which is dependent on the rearrangement of the UBE3A (Ubiquitin Protein Ligase E3A) gene. A summary of the above is provided in Figure 7.

The current landscape of the KD includes modifications to the CKD and is expanding to increasingly younger patient groups, with the possibility of applying the KD from the neonatal period to late old age (Table 4). In addition to drug-resistant epilepsy, which is still the leading indication for KD implementation, new neurological and non-neurological indications are emerging. In addition, its very high effectiveness and applicability in the most severely ill patients in intensive care units using parenteral nutrition in cases of NORSE/FIRES (new-onset refractory status epilepticus/febrile infection-related epilepsy syndrome) have been recognized. Recent advances in personalized medicine prove that genetic, metabolomic, and microbiomic profiling can significantly influence patient selection and significantly optimize KD-based dietary interventions. Molecular high-throughput studies and a multiomic approach have identified key metabolic pathways influencing the response to the KD, enabling the further design of individualized treatment strategies. Currently, the key goal in designing ketogenic therapy should be the integration of genomic, metabolomic, and microbiomic data to develop biomarker-based nutritional protocols, the determination of which will contribute to greater efficacy and safety in the use of the KD.

### 5.6. Critical Methodological Assessment of the Cited Studies

Methodological assessment of the studies referred to reveals a number of limitations that need to be taken into account in the interpretation of the findings. The studies are frequently characterized by small patient groups, e.g., comprising only 16 patients [63], 11 patients [85], or 17 subjects in the study group and 21 in the control group [86]. Human experimental trials also employed small sample sizes, e.g., 29 participants in three study arms, with the trial lasting only 10 days [116]. Such limitations severely reduce statistical power, increase the threat of random error, and rule out the generalizability of results to a larger population.

Another problem is the non-uniform selection of groups and populations, e.g., studies conducted exclusively on infants [41] or with the involvement of subjects of one sex only—males [95] or females [118]. This approach limits the possibility of extrapolation of the results to the entire population and reduces their clinical significance.

Animal models were employed in some of the cited research [43,95,118]. Although these allow examination of biological mechanisms under controlled conditions, they do not reflect the entirety of metabolic and clinical responses in humans. The authors themselves acknowledge the need for more translational research in patients [43].

Although the above studies provide valuable data on the alleged effects and mechanisms of action of the ketogenic diet, methodological limitations, including small and selective sample sizes, short intervention durations, and the use of animal models, undermine the clarity of results and the ability to draw firm clinical conclusions. Therefore, larger, heterogeneous population studies conducted over a long period are necessary to allow for a legitimate conclusion about the efficacy and long-term safety of the ketogenic diet for different patient populations.

## 6. Summary

Metabolomics has transformed our understanding of the ketogenic diet (KD) from a descriptive dietary intervention into a mechanistically defined therapy. The KD represents a complex therapeutic tool with proven efficacy in refractory epilepsy and growing potential in the management of neurodegenerative, metabolic, and psychiatric disorders. By profoundly reshaping cellular and systemic metabolism—including lipid and amino acid pathways, ketone body fluxes, and mitochondrial function—the KD exerts effects that extend beyond energy provision to encompass signaling, epigenetic regulation, and redox homeostasis.

Recent high-throughput metabolomic studies (LC-MS, GC-MS, NMR), in vivo MRS/MRI, and C^13^-based tracing have been pivotal in uncovering these mechanisms, linking specific metabolic shifts—such as enhanced fatty-acid β-oxidation, altered BCAA and kynurenine metabolism, and elevated cerebral glutathione—with clinical benefits including seizure reduction, improved insulin sensitivity, and modulation of neuroinflammation. At the same time, metabolomic analyses underscore potential trade-offs, such as dyslipidemia, micronutrient deficiencies, hepatic and renal stress, and decreased bone mineral density, particularly during long-term interventions or in vulnerable groups such as children, elderly patients, and women of reproductive age.

These insights highlight that KD efficacy and safety are highly individualized and shaped by genetic background, sex, age, dietary composition, and gut microbiome interactions. Large-scale, harmonized high-throughput studies integrating metabolomics, microbiomics, and transcriptomics are essential to establish predictive biomarkers and decision-support tools for precision ketogenic therapy. Future research should also explore ketomimetic strategies capable of reproducing the metabolic and clinical benefits of the KD with fewer dietary restrictions. Ultimately, integrating metabolomic monitoring into clinical practice will be crucial for advancing the KD as a safe, effective, and personalized therapeutic modality.

## Figures and Tables

**Table 1:** Comparison of a regular diet with a CKD and its variants based on [30].

	Regular Diet	CKD	MAD	LGIT	MCT
fat	20–40%	90%	60–70%	60%	30–60%
protein	10–25%	6–8%	20–30%	20–30%	10%
carbohydrates	45–65%	2–4%	6%	10%	15–19%
fat:protein + carbohydrate ratio	0.2–0.3:1	3–4:1	1:1	1:1	1–2:1

**Table 2:** Examples of the use of liquid chromatography in metabolomic research.

No.	Technique/Matrix	Column/Mobile Phase	Metabolite(s)	Sample Preparation	Source
1	LC-MS/MS QTOF blood	UPLC BEH Amide 5 mM ammonium acetate in water (eluent A) and 5 mM ammonium acetate in acetonitrile/water (95/5, *v*/*v*) (eluent B)	βHB, fatty acids, ETA, DHA	coagulation, centrifugation, serum collection, freezing	[15]
2	RP-HPLC plasma	Hypersil Gold aQ, octadecyl silica A and B were Millipore water and acetonitrile, both containing 0.10% formic acid	carnitines: N(5)-acetylornithine	extraction: with methanol containing 10 mol/L^−1^ ethylparaben, 2 mol/ L^−1^ 1,3-nitro-L-tyrosine, 4 mol L^−1^ d4-succinate; deprotection; centrifugation, evaporation, redissolution in methanol	[16]
3	LC-MS/MS blood	-	lipids, carnitines, amino acids, structural analogues of γ-aminobutyric acid and lactic acid	coagulation (EDTA-2Na), centrifugation, serum collection, freezing	[70]
4	LC-MS/MS serum	C18 Mobile phase A: 0.1% formic acid in water, mobile phase B 0.1% formic acid in methanol	βHB	100 µL serum + 800 µL (methanol: acetone = 7:3 *v*/*v*) and 50 µL IS, centrifuged, lyophilized, frozen, dissolved in 100 µL 10% methanol	[20]
5	LC-MS/MS QTOF brain tissue	Phenomenex Kinetex 2.6 μM F5	DON—glutamine inhibitor	homogenized with 3 M HCl + butanol, carried out in the derivative incubated at 60 °C for 30 min, centrifuged, evaporated, resuspended in 50 µL dH_2_O containing 0.2% formic acid	[21]
6	LC-QTOF-MS hippocampus, frontal cortex, plasma	Acquity UPLC BEH Amide, A: 0.1% formic acid + 10 mM ammonium acetate in 20% acetonitrile B: 0.1% formic acid + 10 mM ammonium acetate in 95% acetonitrile	untargeted analysis > homostrachydrine	mixed with methanol at a ratio of 1:5 (*v*/*v*), internal standard: gabapentin, centrifugation	[71]
7	UPLC-ESI-MS/MS CSF	Waters BEH C18, A: water with 0.1% formic acid B: methanol	pyridoxal phosphate, pyridoxal, vitamin B6, pyridoxamine, pyridoxine acid	deproteinization: acetonitrile/methanol (9:1, *v*/*v*) + 0.1% formic acid, incubation in the dark for 20 min, centrifugation, evaporation in nitrogen 60 °C, reconstitution: 0.1% formic acid	[72]
8	HPLC-TOF-MS plasma hippocampus	Phenomenex Kinetex HILIC, A: 50% acetonitrile with 5 mM acetic acid B: 90% ACN with 5 mM acetic acid; pH 5.8	nucleosides, nucleotides, metabolites of purines and pyrimidines, organic acids and their derivatives, peptides and metabolites related to amino acids	homogenization: sonication in 0.1% NH_4_Oac, modified liquid-liquid extraction (Matyash method)	[73]
9	UPLC-MS/MS CSF	Waters BEH C18, A: water + 0.1% formic acid, B: methanol	pyridoxal-5′-phosphate, pyridoxal, pyridoxine, pyridoxamine, pyridoxine acid	degranulation: 6.3% sulfosalicylic acid and acetonitrile, derivatization: 3 N HCl in n-butanol at 65 °C for 30 min, drying, reconstitution: water/methanol (70:30) SW: d9-pipecolic acid	[74]
10	DI/LC-MS/MS post-mortem brain tissue	Absolute IDQ p180 kit, Biocrates	adenosine monophosphate, o-acetylcholine, L-fucose, isobutyric acid, glycerol	extraction: methanol, sonication, centrifugation, evaporation, reconstitution with phosphate buffer	[75]

DHA—omega 3 docosahexaenoic acid; dH_2_O—distilled water; DI/LC-MS/MS—direct injection liquid chromatography coupled to tandem mass spectrometry; DON—6-diazo-5-oxo-L-norleucine; EDTA-2Na—disodium salt of stannous acid; ETA—omega 3 eicosatetraenoic acid; HCl—hydrochloric acid; HILIC—hydrophilic interaction liquid chromatography; HPLC-TOF-MS—high performance liquid chromatography coupled to tandem mass spectrometry with time-of-flight analyzer; LC-MS/MS—liquid chromatography coupled to tandem mass spectrometry; LC-MS/MS QTOF—liquid chromatography coupled to tandem mass spectrometry with time-of-flight analyzer and quadrupole detector; LC-QTOF-MS—liquid chromatography coupled to mass spectrometry with time-of-flight analyzer and quadrupole detector; UPLC-ESI-MS/MS—ultra-performance liquid chromatography coupled to tandem mass spectrometry with electrospray ionization; CSF—cerebrospinal fluid; UPLC-MS/MS—ultra-performance liquid chromatography coupled to tandem mass spectrometry; RP-HPLC—reversed phase high-performance liquid chromatography.

**Table 3:** Examples of the use of gas chromatography in metabolomic research.

No.	Technique/Matrix	Column	Metabolite(s)	Sample Preparation	Source
1	GC-MS/MS urine	BPX-5	172 metabolites: amino acids, organic acids, fatty acids, carbohydrates, nitrogenous compounds, and polyamines	extraction with extraction solution, centrifugation, drying in a vacuum centrifuge, oximation with methoxylamine hydrochloride in pyridine	[76,77,78]
2	GC-MS CSF	BPX-5	56 metabolites, including glycine, xylose, ketoisocarpronic acid	extraction, derivatization	[79]
3	GC-MS dried blood spot	DB-5MS	glutamine, pyruvic acid, L-serine, oxalic acid, caprylic acid, palmitic acid	6 mm circles of blotting paper were extracted with methanol/chloroform, dried under nitrogen, derivatized with MSTFA from TMCS at 70 °C for 1 h	[80]
4	GC-MS plasma	DB-5MS	phosphate, proline, lactic acid, alanine, glutamate, hexadecanoic acid	extraction: methanol, centrifugation drying under nitrogen, oximation with methoxyamine hydrochloride in pyridine 16 h at room temperature, trimethylsilylation of MTBSTFA with 1% TMCS 1 h at 37 °C	[81]
5	GC-TOF-MS plasma	DB-5MS	proline, glutamate, phenylalanine, methionine, lysine, tryptophan, citric acid, uric acid, cholesterol, palmitate, glucose, myo-inositol, creatinine	extraction, centrifugation, drying, oximation, trimethylsilylation	[82]
6	GC-MS/MS plasma	BPX-5	taurine, quinolinic acid, N-acetylneuraminic acid, catechol	extraction SPME	[83]
7	GC-MS/MS serum	CP-SIL 8 CB	3-hydroxybutyrate, acetoacetate, 2-hydroxybutyrate, 3-hydroxyisobutyrate, acetylglycine, decanoic acid, octanoic acid, isoleucine, adipic acid, uric acid, glyoxylic acid, citric acid, tartaric acid, glucosamine, galactose, mannitol, N-acetyl-lysine, 2-aminopimelanoate, 3-hydroxyanthranilate	separation on ion exchange column (elution with water/ hydrochloric acid/ NH_4_OH), freezing, lyophilization, derivatization with MTBSTFA	[84]

GC-MS/MS—gas chromatography coupled to tandem mass spectrometry; CSF—cerebrospinal fluid; GC-TOF-MS—gas chromatography coupled to tandem mass spectrometry with time-of-flight analyzer; MSTFA—N-methyl-N-(trimethylsilyl)trifluoroacetamide; MTBSTFA—N-tert-butyldimethylsilyl-N-methyltrifluoroacetamide; SPME—solid-phase microextraction; TMCS—trimethylchlorosilane.

**Table 4:** Implementation of the KD in the developmental age population and adults [96,152,153,154,155].

The Landscape of the Classic Ketogenic Diet (CKD) in 2025			
CKD modifications:	Patient groups to CKD:	CKD in other diseases:	CKD in the Intensive Care Unit:
MAD diet MCT diet LGIT diet	newborns and infants metabolic diseases mitochondrial disorders genetic epilepsy hypoxic-ischemic injuries	neurodegenerative diseases (AD, PD, ALS) brain tumors and other cancers autism depression and anxiety disorders insulin resistance, obesity type 2 diabetes, NAFLD, PCOS	refractory status epilepticus (NORSE—new-onset refractory status epilepticus; FIRES—febrile infection-related epilepsy syndrome) parenteral KD
The pleiotropic effect of ketosis involves the expression of genes and cellular pathways regulating inflammation, oxidative stress, immune function, cell membrane physiology, intracellular signaling, and intercellular communication.			

## Abbreviations

The following abbreviations are used in this manuscript:

1H-MRS5-MTHF	proton magnetic resonance spectroscopy5-methyltetrahydrofolate
5-MTHF	5-methyltetrahydrofolate
AcAc	acetoacetate
ACE-III	Addenbrooke’s Cognitive Examination–III scale
acetyl-CoA	acetyl coenzyme A
AD	Alzheimer’s disease
ADCS-ADL	AD Cooperative Study–Activities of Daily Living
ADSL	adenylosuccinate lyase
Akt	protein kinase B
ALS	amyotrophic lateral sclerosis
AMP	adenosine monophosphate
AMPK	AMP-activated protein kinase
anti-PD-1	antibody against programmed cell death protein 1
ASD	autism spectrum disorders
ASL	argininosuccinate lyase
ATP	adenosine triphosphate
Aβ	β-amyloid
BCAA	branched-chain amino acids
CDKL5	cyclin-dependent kinase-like 5 deficiency disorder
CKD	classic ketogenic diet
CRP	c-reactive protein
CSF	cerebrospinal fluid
DEE	developmental and epileptic encephalopathies
DEE-STXBP1	syntaxin-binding protein 1
DI/LC-MS/MS	direct infusion/liquid chromatography–tandem mass spectrometry
DTC	differentiated thyroid carcinoma
dTMP	deoxythymidine monophosphate
FADH_2_	flavin adenine dinucleotide reduced form
FGF21	fibroblast growth factor 21
FIRES	febrile infection-related epilepsy syndrome
GABA	gamma-aminobutyric acid
GCL	glutamate-cysteine synthase
GC-MS	gas chromatography–mass spectrometry
GCS	glycine breakdown system
GLUT1DS	glucose transporter type 1 deficiency syndrome
GRIN2A	glutamate receptor, ionotropic, N-methyl D-aspartate 2A
GSD	glycogen storage disease
GSH	glutathione
GTP	guanosine triphosphate
HbA1c	glycated hemoglobin
HDAC	histone deacetylases
HMG-CoA reductase	3-hydroxy-3-methylglutaryl-coenzyme A reductase
HOMA-IR	homeostatic model assessment of insulin resistance
KCNQ2	potassium channelopathies
KD	ketogenic diet
LC	liquid chromatography
LC-MS	liquid chromatography–mass spectrometry
LDL-C	low-density lipoprotein cholesterol
LGIT	low glycemic index treatment
MAD	modified Atkins diet
MCT	medium-chain triglyceride diet
MCT1	monocarboxylate transporter 1
MCT-KD	ketogenic diet enriched in medium-chain fatty acids
MDA	malondialdehyde
MDS-UPDRS	Movement Disorder Society-Unified Parkinson’s Disease Rating Scale
MMKD	modified mediterranean ketogenic diet
MRS/MRI	magnetic resonance spectroscopy/imaging
MS	multiple sclerosis
MS/MS	tandem mass spectrometry
MTHFD2	methylenetetrahydrofolate dehydrogenase 2
mTOR	mechanistic target of rapamycin
NADH	nicotinamide adenine dinucleotide reduced form
NADPH	nicotinamide adenine dinucleotide phosphate reduced form
NAFLD	non-alcoholic fatty liver disease
NKH	non-ketotic hyperglycinemia
NMDA	N-methyl-D-aspartate
NMR	nuclear magnetic resonance spectroscopy
NORSE	new-onset refractory status epilepticus
NRF2	nuclear factor erythroid 2-related factor 2
OAA	oxaloacetate
PCDH19	protocadherin 19
PCOS	polycystic ovary syndrome
PD	Parkinson’s disease
PDAC	pancreatic ductal adenocarcinoma
PDCD	pyruvate dehydrogenase complex deficiency
PDH	pyruvate dehydrogenase
PI3K	reduced phosphoinositide 3-kinaze
QOL-AD	quality of life in AD
RAIR-DTC	radioiodine-refractory differentiated thyroid carcinoma
ROS	reactive oxygen species
SAM	S-adenosylmethionine
SCN1A	sodium voltage-gated channel alpha subunit 1
SCOT	3-oxoacyl-CoA transferase
SIRT1	Sirtuin 1
SMD	standardized mean difference
SOD	superoxide dismutase
TCA	Krebs cycle
TOF	time-of-flight analyzer
TSC1/TSC2	tuberous sclerosis complex
UBE3A	ubiquitin protein ligase E3A
UCD	urea cycle disorder
UHPLC-MS	ultra-high performance liquid chromatography coupled with mass spectrometry
VLCKD	very low-carbohydrate ketogenic diets
βHB	β-hydroxybutyrate
